# Hyaluronic acid reduction-sensitive polymeric micelles achieving co-delivery of tumor-targeting paclitaxel/apatinib effectively reverse cancer multidrug resistance

**DOI:** 10.1080/10717544.2020.1770373

**Published:** 2020-06-03

**Authors:** Xiaoqing Zhang, Xiaomei Ren, Jiayin Tang, Jiangtao Wang, Xiang Zhang, Peng He, Chang Yao, Weihe Bian, Lizhu Sun

**Affiliations:** a Department of Mastopathy, The Affiliated Hospital of Nanjing University of Chinese Medicine (Jiangsu Province Hospital of TCM), Nanjing, China; b The Department of Oncology, The Affiliated Shuyang Hospital of Xuzhou Medical University, Suqian, China

**Keywords:** Apatinib, tumor actively targeted, multidrug resistance, co-delivery, paclitaxel

## Abstract

Multidrug resistance (MDR) of cancer cells is a significant challenge in chemotherapy, highlighting the urgent medical need for simple and reproducible strategies to reverse this process. Here, we report the development of an active tumor-targeting and redox-responsive nanoplatform (PA-ss-NP) using hyaluronic acid-*g*-cystamine dihydrochloride-poly-ε-(benzyloxycarbonyl)-L-lysine (HA-ss-PLLZ) to co-deliver paclitaxel (PTX) and apatinib (APA) for effective reversal of MDR. This smart nanoplatform specifically bound to CD44 receptors, leading to selective accumulation at the tumor site and uptake by MCF-7/ADR cells. Under high concentrations of cellular glutathione (GSH), the nanocarrier was degraded rapidly with complete release of its encapsulated drugs. Released APA effectively inhibited the function of the P-glycoprotein (P-gp) drug pump and improved the sensitivity of MDR cells to chemotherapeutic agents, leading to the recovery of PTX chemosensitivity in MDR cells. As expected, this newly developed intelligent drug delivery system could effectively control MDR, both *in vitro* and *in vivo*.

## 1. Introduction

Chemotherapy remains a major modality of cancer treatment. However, its efficacy is largely hindered by multidrug resistance (MDR) (Mi et al., 2010; Sivak et al., 2017). MDR occurs as a result of tumor cells adapting to various stimuli driven by complex mechanisms and the underlying pathways are still not clearly understood. The main mechanism of MDR constitutes overexpression of cell membrane-bound ATP-binding cassette (ABC) transporters, including P-glycoprotein (P-gp), MDR1, ABCG2, and MPR1 (Allen & Schinkel, 2002; Austin & Ross, 2003^;^ Lee, 2004). These proteins facilitate the transport of chemotherapeutic drug molecules out of cells, leading to reduced intracellular anticancer drug concentrations and, eventually, decreased effectiveness (Allen & Schinkel, 2002; Austin & Ross, 2003; Lee, 2004).

Combination therapy with different drugs plays a major role in increasing the efficacy of tumor therapy. Substantial evidence suggests that co-administration of multiple drugs can effectively increase anticancer efficiency, decrease side-effects, and combat MDR (Kemp et al., 2016; Li et al., 2016; Upponi et al., 2018). Paclitaxel (PTX), a traditional chemotherapeutic agent, is widely applied for treatment of various cancers, including breast, lung, and ovarian tumor types (Sofias et al., 2017). However, when used alone, its efficacy is significantly limited by poor solubility and toxic side-effects (Huang et al., 2018). Apatinib (APA), a tyrosine kinase inhibitor (TKI) and anti-neoplastic drug, is clinically used to treat colon cancer, non-small cell lung cancer, and gastric carcinoma (Li et al., 2012; Geng & Li, 2015). The drug competes with ATP in binding to ATP sites of the catalytic domains of several oncogenic tyrosine kinases, leading to inhibition of angiogenesis, metastasis, invasion, and proliferation (Billah et al., 2011). Recently, APA was shown to effectively overcome MDR by exerting suppressive effects on P-gp function (Mi et al., 2010; Wei et al., 2018; Xu et al., 2019). For example, the group of Zhou demonstrated remarkable APA-induced DOX accumulation in MCF-7/ADR cells through suppression of P-gp activity (He et al., 2019). Moreover, Xu et al. (2019) showed that APA could enhance the chemosensitivity of gastric cancer to PTX, both *in vitro* and *in vivo*, with limited side-effects. However, clinical trials have revealed several side-effects of APA treatment, including hand-foot syndrome, proteinuria, and hypertension (Dai et al., 2017). Thus, the risks associated with co-administration of APA and PTX include serious side-effects and low efficacy due to differences in pharmacokinetics, biodistribution, and membrane transport (Liang et al., 2013; Jang et al., 2016). Here, we aimed to develop a simple, economical and industrializable formulation for co-delivering PTX and APA, which can achieve effective reversal of MDR with acceptable side-effects.

Currently, polymeric micelles are extensively used to deliver multiple drugs owing to their significant advantages over administration of free drugs (Kemp et al., 2016; Li et al., 2016; Upponi et al., 2018). Numerous encapsulated agents within a single platform can eliminate the differences in pharmacokinetics and distribution of multiple drugs, leading to eventual improvement of combined therapeutic efficacy (Hu et al., 2016). Additionally, polymers are internalized by cancer cells through nonspecific or specific endocytosis, allowing bypass of ABC transporters and, consequently, increased intracellular drug concentrations for MDR cancer cell killing (Shi et al., 2015; Gupta et al., 2017) Modified ligands (such as peptides, antibodies, aptamers, folic acid, and hyaluronic acid (HA)) on surfaces of nanomedicines are also able to accurately target tumors and enhance cell uptake. Once internalized via receptor-mediated endocytosis, these molecules enhance the anti-MDR effect (Srinivasarao & Low, 2017). Controlled drug release by stimuli-responsive polymeric micelles in reponse to stimulatory factors in the tumor microenvironment, such as enzymes, redox, and pH, has been achieved, leading to significant reduction of side-effects of anti-tumor drugs (Wei et al., 2017; Sun et al., 2019). The tumor cytoplasmic level of reductive glutathione (GSH, 2–10 mM) is 100–1000-fold that in normal cells, (Jiang et al., 2017) which facilitates drug release by redox-responsive polymeric micelles into cancer cells (Li et al., 2019).

Hyaluronic acid (HA), a natural acidic polysaccharide macromolecule, is widely used for drug delivery owing to its biodegradable and nontoxic nature, along with excellent biocompatibility and easy chemical modification (Huang & Huang, 2018). HA-binding receptors, such as RHAMM and CD44, have been shown to be overexpressed on various cancer cell surfaces, further expanding the broad applicability of HA-based nanomedicines for active tumor-targeted drug delivery (Jiang et al., 2012). Additionally, HA exhibits low accumulation in liver because of weak specificity to its binding site in this organ, thereby promoting accumulation of HA-based nanomedicines in tumor tissue (Hwang et al., 2017; Souchek et al., 2018). Multiple stimuli-responsive nanomedicines based on HA have been developed for cancer therapy, such as an enzyme-responsive HA-based nanogel for drug delivery that is preferentially internalized by CD-44-overexpressing cancer cells (Yang et al., 2015).

**Scheme 1.**
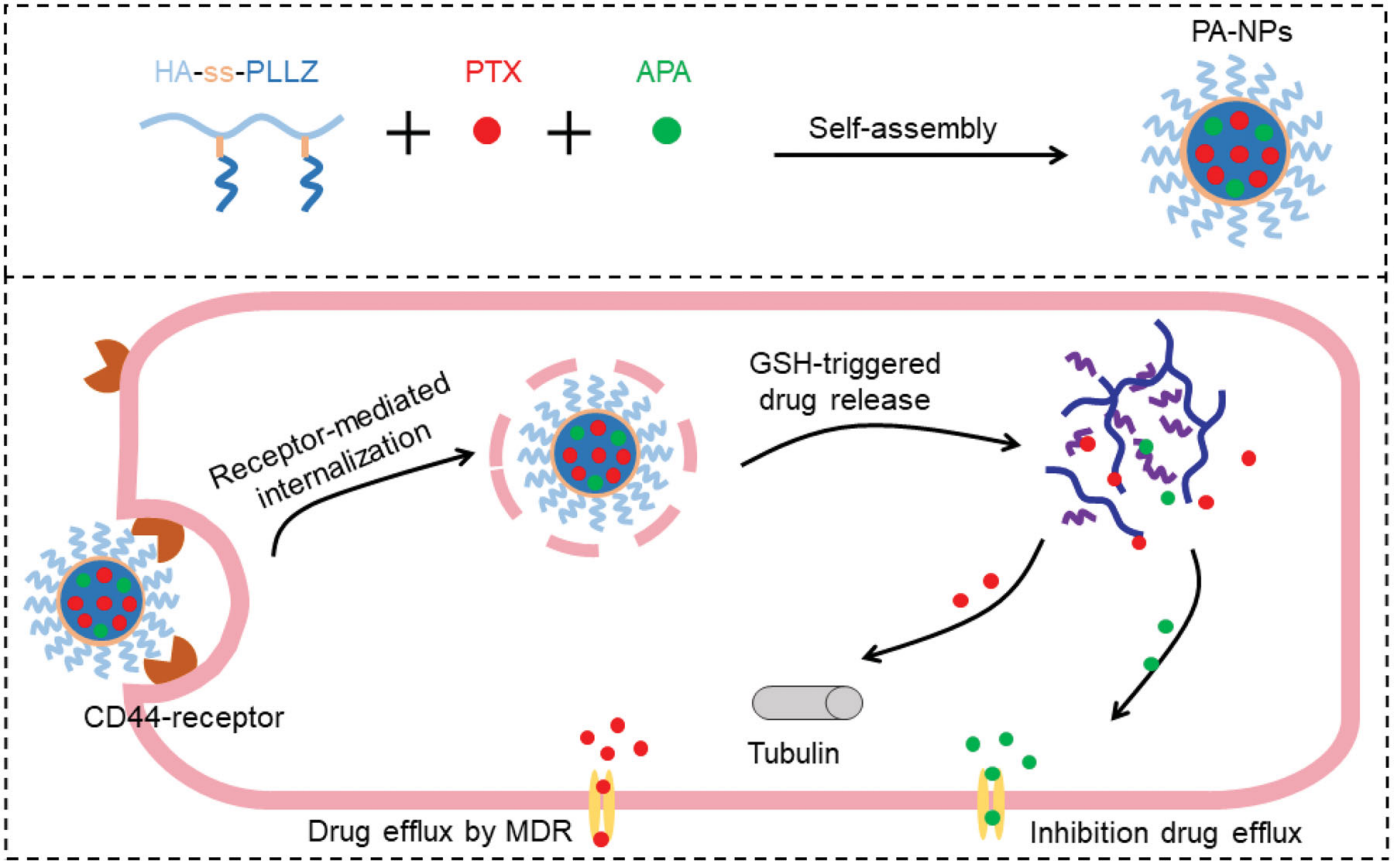
Preparation and application of PTX and APA co-loaded micelles and GSH-triggered drug release in tumor cells. Firstly, PTX, APA, and HA-ss-PLLZ were assembled with polymer micelles (PA-NPs) in aqueous solution. Tumor cells were targeted with PA-NP micelles via *i.v.* injection. Following internalization into cancer cells, PTX and APA were rapidly released from PA-NPs under conditions of high GSH levels. APA inhibited P-gp activity, leading to elevated amounts of PTX within cells and, ultimately, reversal of MDR.

In this study, we designed active tumor-targeting and redox-responsive hyaluronic acid-*g*-cystamine dihydrochloride-poly-*ε*-(benzyloxycarbonyl)-L-lysine (HA-ss-PLLZ) drug-releasing micelles (PA-NPs) encapsulating paclitaxel (PTX) and apatinib (APA), with a view to combating MDR (Scheme 1). PA-NPs could specifically target cancer cells and achieve delivery of high intracellular concentrations of drugs, followed by complete release of PTX and APA in response to high levels of GSH in tumor cells to facilitate MDR cell destruction through synergistic effects. The efficacy of PA-NPs in MDR cancer treatment was investigated both *in vitro* and *in vivo*.

## 2. Materials and experiments

### 2.1. Synthesis of HA-ss-PLLZ

Hyaluronic acid-*g*-cystamine dihydrochloride-*β*-benzyl-L-aspartate (HA-ss-PLLZ) was synthesized in two steps. Firstly, cystamine dihydrochloride (Cys) was reacted with the carboxyl group on the side of HA to obtain HA-*g*-Cys. HA-ss-PLLZ was generated via ring-opening polymerization of *N*ε-benzyloxycarbonyl-L-lysine-*N*-carboxyanhydride (Lys-NCA) according to previous reports, with HA-*g*-Cys serving as a macroinitiator (Xu et al., 2018).

Briefly, 35.0 mL dry formamide containing 600.0 mg HA (0.2 mmol), 25.3 mg NHS (2.2 mmol), and 115.2 mg EDC (0.6 mmol) was stirred at 30 °C for 2 h. Next, 25.0 mL Cys (88.0 mg, 0.4 mmol) in dry formamide was mixed with the above solution and further incubated overnight at 30 °C under a stream of nitrogen. Products were purified via dialysis (MWCO, 1000 Da) against water for two days and freeze-dried to acquire the final product, HA-*g*-Cys.

For preparation of HA-ss-PLLZ, 10.0 mL dry formamide-containing 260.0 mg Lys-NCA (0.8 mmol) was mixed with 320.0 mg HA-*g*-Cys (0.1 mmol) in 45.0 mL dry formamide under dry argon and reacted for 72 h under dry argon conditions at 40 °C. The final polymer, HA-ss-PLLZ, was obtained through lyophilization after purification by dialysis against distilled water.

### 2.2. Micelle preparation

PTX and APA co-loaded micelles formed using HA-ss-PLLZ, PTX, and APA were designated PA-NPs, PTX- loaded micelles formed using HA-ss-PLLZ and PTX denoted P-NPs, and coumarin-6-loaded blank micelles formed using HA-ss-PLLZ and coumarin-6 abbreviated as C-NPs. All micelles were prepared with the coprecipitation method. Briefly, PTX (4.0 mg), APA (0.5 mg), and HA-ss-PLLZ (20.0 mg) were dissolved in 2.0 mL DMSO under ultrasonic conditions and added dropwise into 10.0 mL PBS under vigorous stirring for 5 h. DMSO was removed by dialyzing (MWCO: 3,500 Da) against deionized water at 4 °C for 12 h. Subsequently, unloaded PTX and APA in micelle solutions were filtered out through 0.45 µm microporous membranes to obtain PA-NPs.

The concentrations of PTX and APA in PA-NPs were determined using high-performance liquid chromatography (HPLC). The encapsulation efficiency (EE) and loading capacity (LC) of PTX and APA were calculated as follows:

$$\mathrm{LC}\;(\%)\;=\;\frac{\text{weight of the drug in the micelles}}{\text{weight of the whole micelles}}\;\times 100\%$$

$$\mathrm{EE}\;(\mathrm{wt}\%)\;=\;\frac{\text{weight of the drug in the micelles}}{\text{weight of feed drug}}\;\times 100\%$$

### 2.3. *In vitro* redox-triggered disassembly and drug release

To investigate redox-triggered micelle disassembly, size changes in PA-NPs micelles under various GSH concentrations were determined using dynamic light scattering (DLS). Briefly, 4.0 mL ss-NP nanoparticle solutions with different concentrations of GSH (0, 10 µM, and 10 mM) were placed in an incubator and shaken at 100 rpm (37 °C). PA-NP sizes were measured via DLS at the predetermined time-points. After culturing under 10 mM GSH, PA-NP structures were examined using transmission electron microscopy (TEM).

Dialysis was employed to study GSH-triggered release of PTX and APA from PA-NP micelles. Typically, 2.0 mL PA-NP micelles were subjected to dialysis (MWCO: 3,500 Da) and incubated in 48.0 mL release medium (PBS: 0.1 M, pH 7.4; Tween 80: 0.1% w/v; GSH: 0, 10.0 µM, or 10 mM) at 37 °C under a nitrogen atmosphere. At various time-points, 200 µL buffer solution was collected and replaced with 200 µL fresh release buffer. The amounts of released PTX and APA were measured with the aid of HPLC.

### 2.4. Cellular uptake analysis

Competitive experiments were employed to investigate the HA-mediated active targeting properties of our newly designed therapeutic nanoparticles. Typically, MCF-7/ADR cells (1.0 × 106 per well) seeded on six-well plates were cultured in 10.0 mg/mL HA for 3 h, followed by incubation with C-NPs (coumarin-6, 400.0 ng/mL) for 4 h. Cells were washed with pre-cold PBS, fixed in 4% paraformaldehyde solution, stained with DAPI, and observed under a fluorescence microscope (FM, Leica Microsystems, Wetzlar, Germany). Non-HA-pretreated cells were used as the control group.

For quantitative analysis of intracellular coumarin-6 concentrations, MCF-7/ADR cells were precultured with or without free HA, followed by treatment with C-NPs (coumarin-6, 400.0 ng/mL) for 4 h. Cells were washed, trypsin-digested, collected, and analyzed on a BD FACSCalibur flow cytometer (FCM).

### 2.5. *In vitro* cytotoxicity analysis

#### 2.5.1. Optimization of the combination ratio of APA and PTX

To achieve a maximal combination effect, the optimal combination ratio between APA and PTX was initially established. In brief, 5000 MCF-7/ADR cells were seeded in each well of 96-well plates and cultured for 24 h under normal culture conditions. Next, cells were treated with various concentrations of drugs (APA, PTX, and APA combined with PTX at molar ratios of 10/1, 8/1, 4/1, 2/1, 1/1, 1/2, 1/4, 1/8, and 1/10) for 48 h, followed by the addition of 20.0 μL MTT solution (5.0 mg/mL in serum-free RPMI 1640) to each well. After incubation for 4 h, the solution was replaced with 200.0 μL DMSO and absorbance determined on a microplate reader. GraphPad Prism 6 was applied to calculate the inhibitor concentration (IC_50_) value for each drug. The combination index (CI) of APA and PTX against MCF-7/ADR cells was determined according to the formula: [Li et al., 2020] CI = $\frac{D1\chi }{D1}+\;\frac{D2\chi }{D2},$ whereby D1 and D2 represent IC_50_ values of APA and PTX alone and *D*1χ and *D*2χ represent molar ratios of APA and PTX in the combination group at IC_50_ values. Values of CI <1 indicate synergistic effects of drugs.

#### 2.5.2. MTT study

The cytotoxicities of other formulations against MCF-7/ADR and MCF-7 cells were investigated using the MTT assay. In brief, both cell types (5.0 × 103 cells/well) were seeded on 96-well plates and incubated with various concentrations of PTX, APA, PTX + APA, P-NPs, and PA-NPs for 48 h. Viable cells in each well were detected as described above.

### 2.6. Drug accumulation and efflux assay

#### 2.6.1. PTX accumulation and efflux study

MCF-7 and MCF-7/ADR cells (1.0 × 10^5^ cells per well) seeded on 12-well plates were treated with PTX, PTX + APA, P-NPs, and PA-NPs (equivalent to 10.0 µg/mL PTX and 2.0 µg/mL APA) for 1, 2, and 4 h, respectively. Cells were collected, washed, and degraded with lysis buffer. PTX was extracted by mixing acetonitrile and cell lysates (v/v = 2:1) under ultrasonication, followed by centrifugation (4 °C, 15 min, 10^4^ rpm). PTX levels in the supernatant fractions were determined using HPLC. The PTX contents determined were normalized to protein levels in cell lysates.

For the PTX efflux study, cells were pre-incubated with PTX, PTX + APA, P-NPs, or PA-NPs for 4 h. After incubation, cells were rinsed three times with PBS and further cultured in RPMI 1640 for 1, 2, or 4 h. Intracellular PTX concentrations were measured using HPLC as described above.

#### 2.6.2. Effects of APA on Rh123 accumulation

Cells (1.0 × 10^6^ cells per well) seeded on six-well plates were pretreated with APA, PTX, APA + PTX, P-NPs, and PA-NPs (equivalent to 2.0 μg/mL APA) for 3 h, followed by culturing with Rh123 (5.0 μg/mL) for 4 h. Fluorescence of Rh123 in cells was measured via FM and FCM. Verapamil (VRP), a first-generation P-gp inhibitor, was employed as the positive control (2.0 μg/mL).

### 2.7. Pharmacokinetic and biodistribution assessments

In the pharmacokinetic study, SD rats were administered PTX, P-NPs, or PA-NPs (fixed PTX dose of 6.0 mg/kg) via tail vein injection. At predetermined time intervals (0, 0.08, 0.25, 0.5, 1, 2, 4, 8, 12, and 24 h), blood (500 μL) was collected from the orbital and plasma obtained by centrifugation. Subsequently, plasma was mixed with acetonitrile (1.0 mL) and centrifuged (15 min, 10^4^ rpm) to extract drugs. The supernatant was subsequently collected and dried under nitrogen. Finally, the residues obtained were redissolved in acetonitrile for HPLC analysis.

In the biodistribution study, an MCF-7/ADR tumor-bearing mouse model was established by injecting 4.0 × 10^6^ MCF-7/ADR cells into the mammary fat area of individual mice. Animals were administered PTX or PA-NPs (6.0 mg/kg PTX) via tail vein injection. At 24 h post-injection, major tissues (tumor, kidney, lung, spleen, liver, and heart) were dissected after sacrifice. Tissues were weighed, homogenized, and centrifuged to extract PTX and concentrations detected according to the protocols specified above.

### 2.8. *In vivo* antitumor efficacy

MCF-7/ADR cells (1.0 × 10^6^ cells per mouse) were injected into the mammary fat area. Mice were randomly assigned to six groups (*n* = 6) when tumor sizes reached ∼80 mm^3^. Each mouse was injected with saline, PTX, APA, PTX + APA, P-NPs, or PA-NPs at a PTX dose of 10.0 mg/kg on days 0, 3, and 6 (the first drug administration was recorded as day 0). Body weights and tumor sizes of mice were monitored every 3 days for 21 days after the first drug treatment. Tumor volumes (V) were calculated based on the equation: V (mm^3^) = 1/2 × (length × width Sivak et al., 2017). Tumor-bearing mice were euthanized on day 21 and major tissues (spleen, tumor, liver, lung, heart, and kidney) collected, followed by staining with hematoxylin and eosin (H&E). The tumor inhibition rate (TIR) was calculated according to the following equation: TIR (%) = (*V*saline − *V*sample)/*V*sample, where *V*saline and *V*sample represent the tumor volume of Saline group and drug treated groups at day 21, respectively.

### 2.9. Statistical analysis

All quantitative data are presented as means ± SD. Student’s *t*-test and ANOVA were applied to analyze the differences between two groups.

## 3. Results and discussion

### 3.1. HA-ss-PLLZ characterization

**Figure 1.**
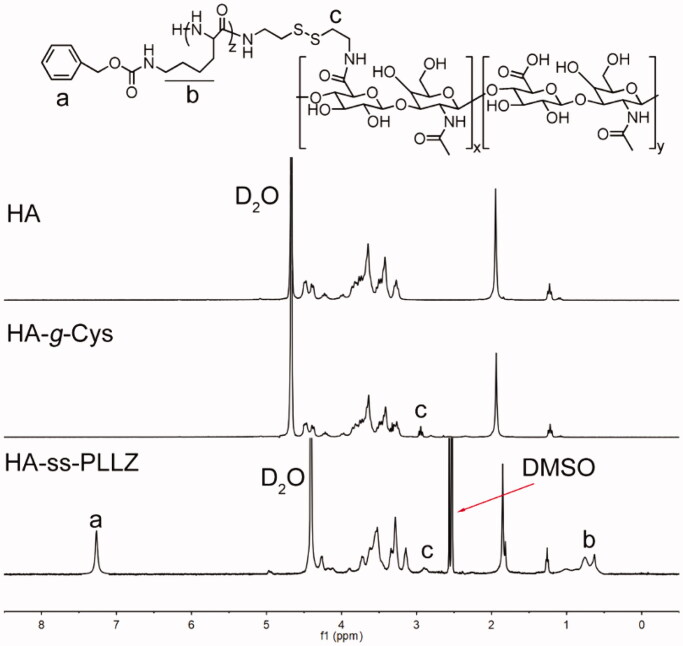
^1^H NMR spectra of HA and HA-*g*-Cys in D_2_O and HA-ss-PLLZ in DMSO-*d6*.

Details of the synthesis route of the redox-sensitive polymer, HA-ss-PLLZ, are presented in Supplementary Figure S1. Polymers with low critical micelle concentrations (CMC) may overcome the effects of hemodilution, thereby avoiding premature drug leakage in the blood circulation (Hu et al., 2016; Zhang et al., 2020). Moreover, an appropriate size may be required to maximize nanomedicine accumulation in tumor sites mediated by the enhanced permeability and retention (EPR) effect (Kinoshita et al., 2017). To achieve low CMC and optimal sizes of HA-ss-PLLZ-formed micelles, a series of HA-ss-PLLZ with different molar ratios of HA and PLLZ were prepared. The structures of the polymers were confirmed using gel permeation chromatography (GPC) and proton nuclear magnetic resonance (^1^H NMR) spectroscopy. The Nile Red assay was performed to assess the CMC values of these polymers. The sizes of polymeric micelles were determined using DLS. As shown in Supplementary Table S1, HA-ss-PLLZ containing ∼20 PLLZ (HA-ss-PLLZ_20_) displayed the lowest CMC value (6.1 µg/mL) and small size (about 90 nm) with a low polydispersity index (PDI). Accordingly, HA-ss-PLLZ_20_ (referred to in subsequent sections as HA-ss-PLLZ) was employed for subsequent experiments. ^1^H NMR spectra of HA, HA-Cys, and HA-ss-PLLZ with assignment of characteristic proton peaks are presented in Figure 1. The peak at 2.9 ppm was attributed to Cys, signals at 3.2–4.8 ppm to HA, and signals at 0.5–1 ppm and 6.8–7.2 ppm to PLLZ. Our collective data support the successful synthesis of HA-ss-PLLZ.

The biocompatibility of nanocarriers, an important feature for drug delivery, is mainly reflected in two aspects: (1) cytotoxicity against normal and cancer cells and (2) influence on stability of the red blood cell (RBC) membrane. The biocompatibility of HA-ss-PLLZ was determined via MTT and hemolysis assays. Two breast cancer cell lines (MCF-7/ADR and MCF-7) and a normal breast cell line (MCF-10A) were employed to evaluate cytotoxicity of the HA-ss-PLLZ polymer (Supplementary Figure S2A). The viability of all three cell lines was >90% after incubation with HA-ss-PLLZ for 48 h within a polymer range of 0.1–10 mg/mL, suggestive of good efficacy. Similarly, hemolysis ratios of HA-ss-PLLZ were lower (5%) within a range of 0.1–10 mg/mL (Supplementary Figure S2B), indicating no unacceptable effects on the RBC membrane. The results obtained validate the biocompatibility of HA-ss-PLLZ.

### 3.2. PA-NP characterization

**Figure 2.**
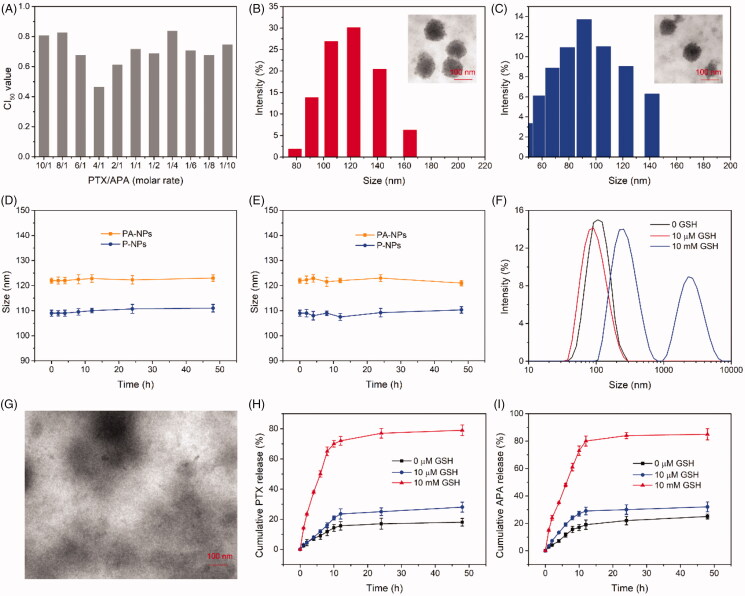
Characterization of PA-NPs and P-NPs. (A) CI_50_ values of different combination ratios of PTX and APA against MCF-7/ADR cells after 48 h treatment. (B,C) TEM imaging and size distribution of PA-NPs (B) and P-BPs (C). (D, E) Stability of PA-NPs and P-NPs in PBS (D) and PBS containing 20% FBS (E) (*n* = 5). (F) Size changes in PA-NPs after incubation for 8 h in PBS with 0 µM, 10 µM, and 10 mM GSH. (G) TEM images of PA-NPs after treatment with 10 mM GSH for 8 h. (H,I) Cumulative release of PTX (H) and APA (I) from PA-NPs incubated with 0 µM, 10 µM GSH, and 10 mM GSH (*n* = 3).

The optimal combination ratio of APA and PTX against MCF-47/ADR cells was determined before drug-loaded micelle preparation. Typically, cells were treated with APA/PTX (A/P) at different molar ratios and 50% combination index (CI_50_) calculated (Figure 2(A)). CI > 1, CI = 1, and CI < 1 represent antagonistic, equivalent, and synergistic effects of the combined drugs (Li et al., 2020). At a molar ratio of 4:1, the CI_50_ value was lowest, indicative of maximal cell growth inhibition. Accordingly, the optimal APA: PTX molar ratio was selected as 4: 1 for further experiments.

Subsequently, PTX-loaded and PTX/APA co-loaded micelles (designated P-NPs and PA-NPs, respectively) were generated using the coprecipitation method. P-NPs and PA-NPs displayed small sizes of 122.3 ± 3.1 nm and 109.2 ± 1.9 nm, respectively, along with narrow size distribution (Figure 2(B,C) and Supplementary Table S2). Zeta potential values of PA-NPs and P-NPs were −15.3 mV and −16.2 mV, respectively (Supplementary Table S2). PA-NP and P-NP structures were spherical with good monodispersity, as determined using transmission electron microscopy (Figure 2(B,C)). In addition, PA-NPs displayed a high capacity for PTX and APA uptake, with drug loading efficiencies (LC) of 13.5 and 3.2%, respectively (Supplementary Table S2). Data from the stability assay showed no significant alterations in the sizes of both PA-NPs and P-NPs in PBS with or without 20% FBS, suggesting high stability of these micelles in the blood circulation (Figure 2(D,E)).

### 3.3. Micelle disassembly and drug release triggered by GSH

To examine the process of GSH-responsive micelle disassembly, size changes in PA-NPs after treatment with GSH at different concentrations and times were detected via DLS. As shown in Figure 2(F), stability of PA-NPs in PBS was maintained for over 8 h in the absence of GSH. Upon treatment with 10 µM GSH (extracellular level) for 8 h, the sizes of PA-NPs were not significantly altered. However, as the GSH concentration increased to 10 mM (intracellular level), PA-NP sizes increased from 100 to 650 nm and PDI from 0.21 to 0.86 (data not shown). TEM imaging further revealed rapid disintegration of PA-NPs in 10 mM GSH (Figure 2(G)). Our data suggest that PA-NPs are stable in the blood circulation and extracellular conditions but quickly disassemble under conditions of high intracellular GSH.

The dialysis method was employed to examine GSH-responsive drug release of PA-NPs. As presented in Figure 2(H,I), in the absence of GSH, PA-NPs exhibited negligible PTX and APA release (both <25%) within 48 h, indicative of good nanoparticle stability. Notably, ∼28% PTX and 32% APA were released from PA-NPs incubated with 10 µM GSH for 48 h. Furthermore, upon treatment of PA-NPs with 10 mM GSH, rapid and complete drug release was observed (both >75%). The results collectively demonstrate that PA-NP stability is maintained during blood circulation and PTX and APA are retained without leakage. Once internalized by cancer cells, these micelles rapidly disassemble and release their cargo under conditions of high intracellular GSH.

### 3.4. HA enhances cellular uptake of PA-NPs

**Figure 3.**
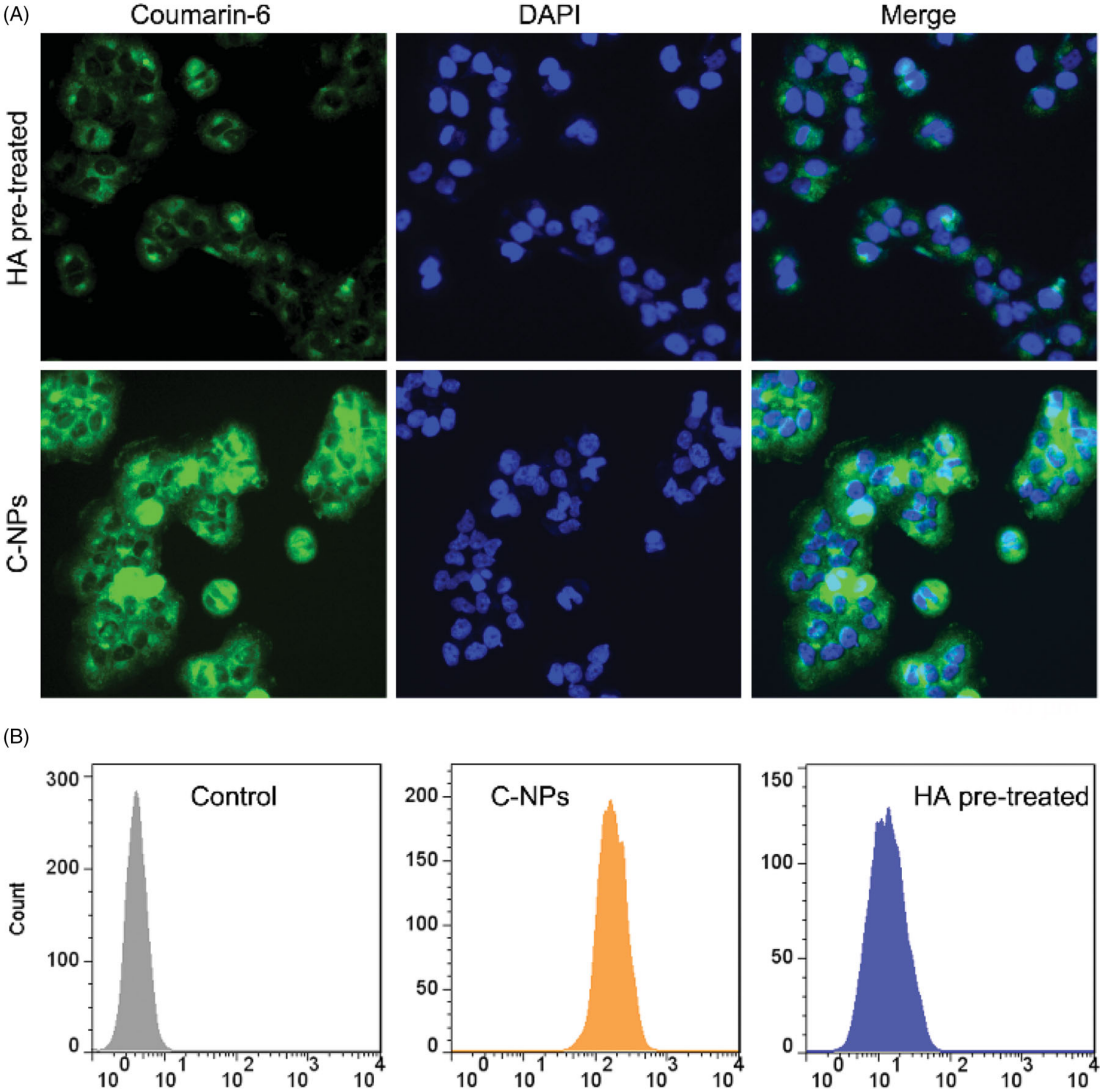
(A) Images of cellular uptake of C-NPs with or without free HA pretreatment. Green and blue fluorescence were observed from coumarin-6 and DAPI, respectively. (B) FCM results on influence of HA on C-NP uptake by MCF-7/ADR cells.

Increased accumulation of therapeutic agents in MDR cells is a key approach for overcoming ABC transporter mediated-MDR. In view of numerous reports that HA specifically targets tumor cells overexpressing the CD44 receptor, including breast and lung cancers (Wu et al., 2017), we hypothesized that HA-coated PA-NPs could significantly accumulate in cancer cells. The effects of HA on cellular uptake of coumarin-6-labeled HA-ss-PLLZ blank micelles (C-NPs) was analyzed quantitatively via flow cytometry (FCM) and qualitatively via fluorescence microscopy (FM) using a high CD44-expressing cell line, MCF-7/ADR (Liu et al., 2018). As shown in Figure 3(A), strong coumarin-6 fluorescence was observed in cells treated with C-NPs for 4 h. However, cellular uptake by free HA pretreated cells was significantly lower. Consistently, FCM data showed strong fluorescence from coumarin-6 in cells treated with C-NPs and weak fluorescence in the HA pretreated group (Figure 3(B)). These results suggest that C-NPs are internalized by MCF-7/ADR cells through a receptor-mediated mechanism. Moreover, HA coating endows C-NPs with high tumor-targeting specificity via CD44 receptor interactions, facilitating enhanced accumulation of therapeutic agents in MDR cells (Huang and Huang, 2018; Yang et al., 2018).

### 3.5. *In vitro* suppression of MDR

**Figure 4.**
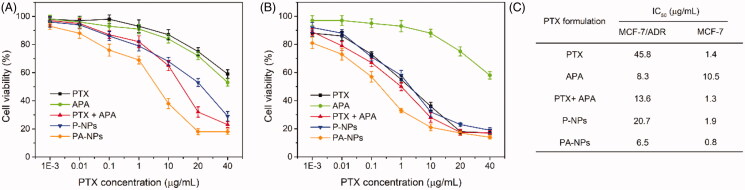
*In vitro* inhibitory activities of all PTX formulations on MCF-7/ADR and MCF-7 cell proliferation measured with the MTT assay. (A, B) Viabilities of MCF-7/ADR cells (A) and MCF-7 cells (B) treated with various concentrations of PTX, APA, PTX + APA, P-NPs, or PA-NPs for 48 h (*n* = 6). (C) IC_50_ values of all drug formulations in both cell lines.

To evaluate the efficacy of the PTX-APA combination in reversal of MDR, the inhibitory effects of PA-NPs on proliferation of MCF-7/ADR and MCF-7 cells were analyzed via MTT. As shown in Figure 4(A–C), PTX showed powerlessly to inhibit the growth of MCF-7/ADR, compared with MCF-7 cells. IC_50_ values of PTX were determined as 45.8 µg/mL in MCF-7/ADR cells and 1.4 µg/mL in MCF-7 cells, confirming significant PTX resistance of MCF-7/ADR cells. Moreover, owing to improved drug delivery and higher resulting intracellular drug concentrations, growth inhibitory effects of PTX on MCF-7/ADR cells were obviously enhanced after encapsulation in micelles formed with HA-ss-PLLZ. However, the IC_50_ value of P-NPs in drug-resistant cells remained markedly higher than that in drug-sensitive cells (20.1-fold). This finding suggests that P-NPs cannot effectively combat MDR based on the nanomedicine-mediated “bypass” effect alone, since drugs released in the cytoplasm risk being pumped out of cells by ABC transporters (Che et al., 2019). Similarly, combination of PTX with DAP induced a significant increase in sensitivity of MCF-7/ADR cells to PTX but did not effectively reverse MDR, which could be attributed to differences in membrane transportation of APA and PTX. Relative to the other treatment groups, PA-NPs markedly inhibited MCF-7/ADR cell growth as a result of increased intracellular PTX accumulation through HA-mediated active tumor targeting and APA-mediated suppression of P-gp function. The IC_50_ values of PA-NPs in MCF-7/ADR cells were 1.3-, 2.5-, and 3.6-fold lower than those of P-NPs, APA + PTX, and free PTX, respectively. Clearly, the sensitivity of MCF-7/ADR cells to PTX was markedly increased by the co-delivery system, facilitating reversal of MDR.

### 3.6. Mechanism of MDR reversal

Data from the MTT assay showed that APA significantly improves the efficacy of PTX against MCF-7/ADR cells. To understand this phenomenon, the mechanism underlying APA-mediated reversal of PTX resistance in MDR cells was further explored. P-gp is known to promote efflux of chemotherapeutic agents out of cancer cells, which enhances MDR. Therefore, inhibition of its expression or function may aid in combating P-gp-mediated MDR. The influence of APA on P-gp expression was initially evaluated (Supplementary Figure S3). Specifically, P-gp levels in MCF-7/ADR cells after treatment with APA, PTX, P-NPs, and PA-NPs were determined via western blot. The P-gp level in MCF-7/ADR cells was markedly higher (about 30-fold) than that in MCF-7 cells, clearly supporting a role in the drug resistance mechanism. Incubation with APA did not significantly affect P-gp expression, indicating that MDR reversal does not involve APA-mediated suppression of P-gp expression, consistent with previous reports (Mi et al., 2010).

**Figure 5.**
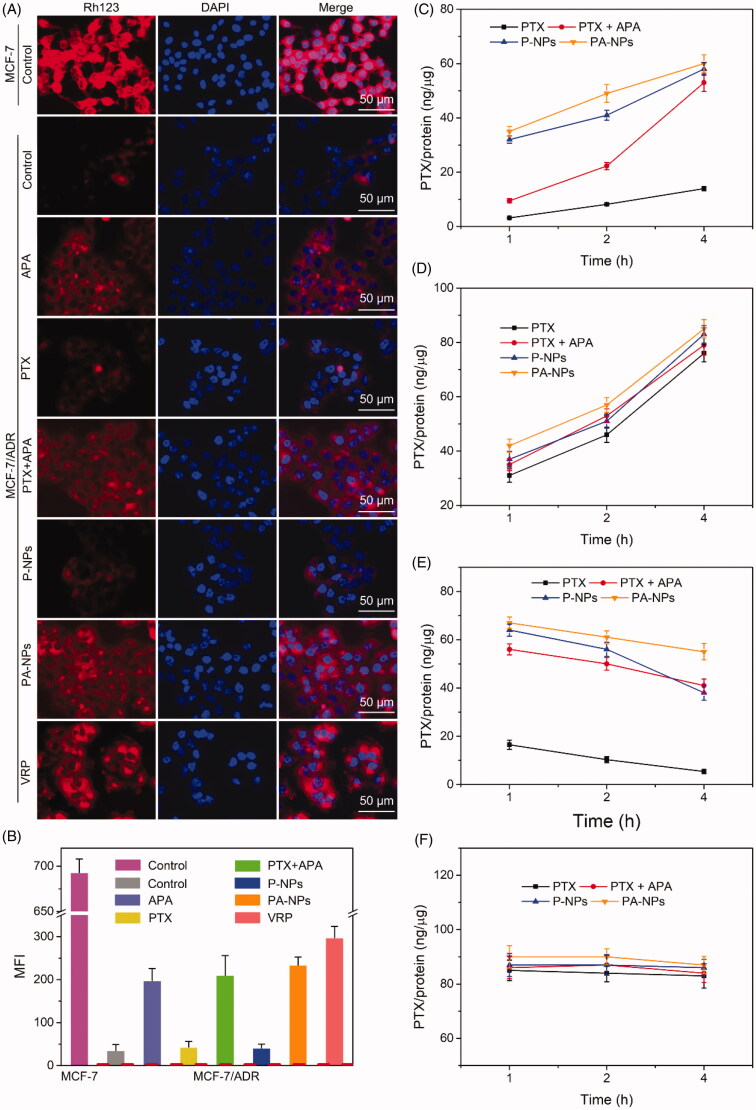
Drug accumulation and efflux assay. (A, B) Cellular uptake of Rh123. MCF-7/ADR cells were pretreated with PBS, APA, PTX, PTX + APA, P-NPs, PA-NPs, and VRP for 3 h, followed by Rh123 for 4 h, and subjected to FM and FCM (*n* = 6). (C, D) PTX accumulation in MCF-7/ADR cells (C) and MCF-7 cells (D) after treatment with PTX, PTX + APA, P-NPs, or PA-NPs for various time periods (*n* = 6). (E, F) PTX efflux from MCF-7/ADR cells (E) and MCF-7 cells (F) after treatment with PTX, PTX + APA, P-NPs, or PA-NPs for various time-periods (*n* = 6).

Subsequently, the influence of APA on P-gp function in membrane transport was analyzed with the aid of drug accumulation and efflux assays. Rh123, a primary substrate of P-gp, was employed to evaluate its membrane transport activity. Verapamil, a first-generation P-gp inhibitor, was used as the positive control. Data are presented in Figure 5(A). As evident from the FM images, the Rh123 signal (red) in MCF-7 cells was markedly higher than that in drug-resistant MCF-7/ADR cells, in keeping with MTT and western blot findings. After pretreatment with free PTX or P-NPs, the Rh123 intensity in MCF-7/ADR cells was not significantly changed, indicating no effect of PTX on internalization of Rh123 into drug-resistant cells. In contrast, the Rh123 signal in MCF-7/ADR cells was significantly increased in APA and PA-NP-preincubated groups, consistent with data from the VRP treatment group. FCM results were consistent with the FM images (Figure 5(B)). Based on the collective data, we suggest that APA promotes intracellular accumulation of P-gp substrate via inhibition of P-gp function to overcome drug resistance.

To further investigate this phenomenon, cellular accumulation and efflux of PTX were examined. In the drug accumulation assay, the concentration and accumulation rates of free PTX in MCF-7/ADR cells were markedly lower than those in MCF-7 cells (Figure 5(C,D)). However, in the combination (PTX + APA) treatment group, levels and accumulation rates of PTX in MCF-7/ADR cells were obviously enhanced. The amounts of PTX detected in MCF-7/ADR cell groups treated with P-NPs and PA-NPs were significantly higher than that of the free PTX treatment group, which was attributed to entry into cells through receptor-mediated endocytic uptake and countering of P-gp mediated PTX efflux. Owing to low endogenous expression of P-gp, drug accumulation patterns in MCF-7 cell groups treated with all the above formulations were not significantly different (Figure 5(D)).

Subsequently, the drug efflux capabilities of low and high P-gp-overexpressing cells were analyzed. To this end, cells were pre-cultured with all PTX formulations for 4 h, followed by incubation in drug-free medium. At the pre-designed time points, intracellular reserves of PTX were measured. As shown in Figure 5(E), in drug-resistant cells, low levels of PTX were retained in the free PTX treatment group and continuously decreased after incubation, which could be explained by the strong efflux effect of P-gp. Interestingly, in the PTX and APA co-treated group, the intracellular PTX level remained elevated with no remarkable subsequent decrease. Similarly, in the P-NP group, the PTX concentration within cells was decreased with increasing incubation time owing to P-gp-mediated efflux. The highest intracellular PTX level was detected in the PA-NP group and showed an insignificant decline. At the beginning of the experiment, the PTX concentration in drug-sensitive cells (Figure 5(F)) was obviously higher than that in drug-resistant cells and displayed no significant changes within 4 h of culture. These results further confirm that APA has the capability to significantly improve drug transport into MCF-7/ADR cells through inhibition of P-gp function, eventually leading to reversal of MDR.

### 3.7. *In vivo* antitumor effects of HA nanomedicines

**Figure 6.**
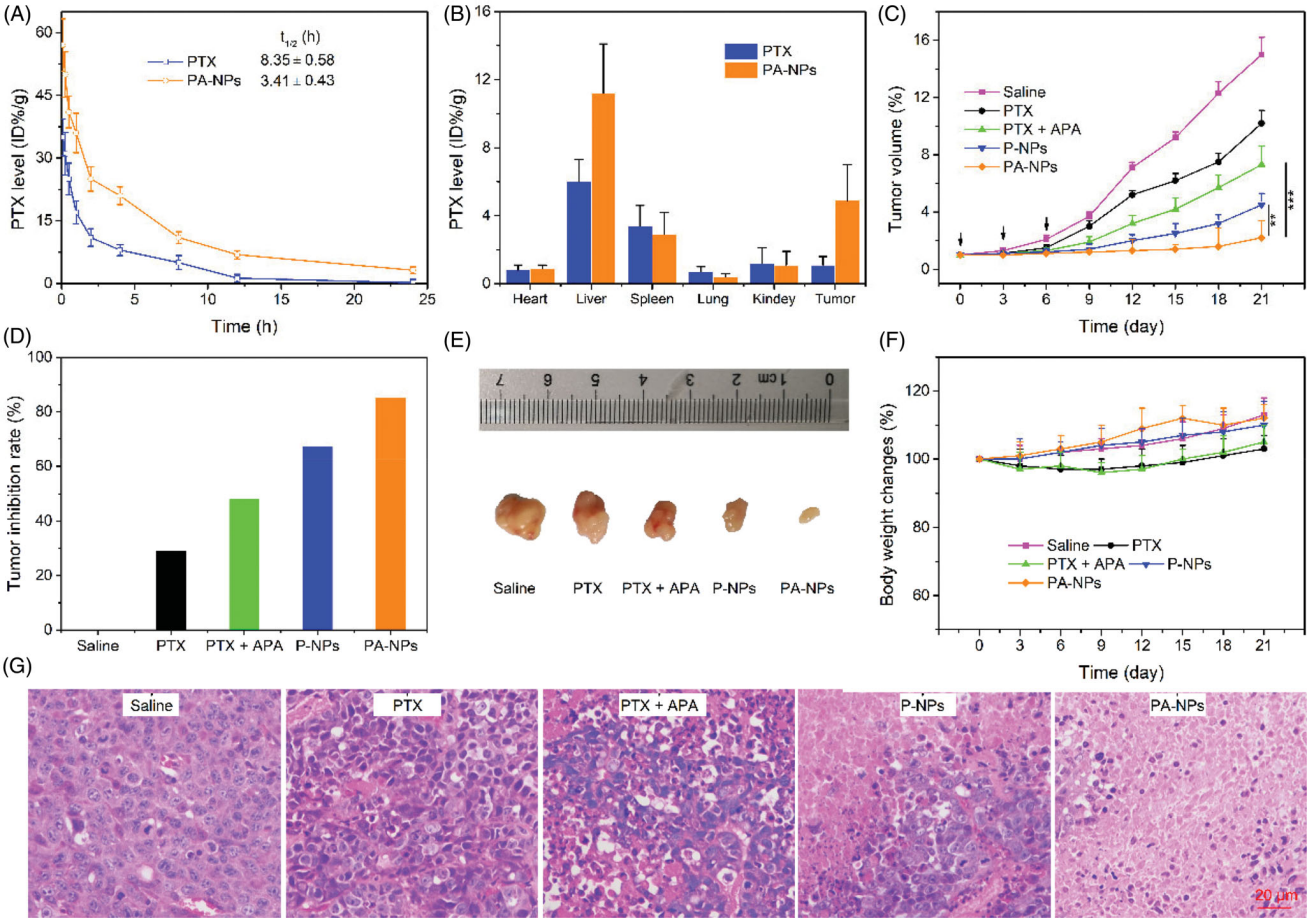
*In vivo* experiments. (A) *In vivo* pharmacokinetics of PTX and PA-NPS in SD rats. The PTX level was calculated as % injected dose per gram of tissue (% ID/g, *n* = 5). (B) Biodistribution of PTX and PA-NPS in MCF-7/ADR tumor-bearing mice. The PTX level was calculated as %ID/g (*n* = 5). (C–F): (C) tumor growth curves; (D) tumor inhibition rate; (E) tumor images; and (F) curve of body weight changes in MCF-7/ADR tumor-bearing mice treated with saline (control), PTX, PTX + APA, P-NPs, and PA-NPs (*n* = 6, **p < .05, ***p < .001). (G) Representative H&E-stained tumor slice images acquired at 20× objective.

Insufficient drug accumulation in tumor tissue can also contribute to resistance, highlighting the necessity for effective targeted delivery of therapeutic drugs to tumors. HA-based nanomedicines are reported to prolong drug circulation in blood, resulting in enhanced drug accumulation in tumor tissue through HA-mediated active targeting (Uthaman et al., 2018). This phenomenon was confirmed via pharmacokinetics and biodistribution assays. Briefly, SD rats were administered PTX and PA-NPs (6 mg PTX/kg) through tail vein injection and plasma concentrations of PTX detected via HPLC (Figure 6(A)). Compared with free PTX, PA-NPs exhibited longer blood circulation times with an elimination half-life of 8.35 h, which was 2.4-fold that of the free PTX group. After 24 h, the amount of PTX in tumor tissue in the PA-NP group was 7.6-fold higher than that of the free PTX treatment group (Figure 6(B)), clearly demonstrating prolonged blood circulation and enhanced tumor-targeted PTX accumulation of PA-NPs.

The MCF-7/ADR mouse tumor model was employed to investigate the *in vivo* therapeutic efficacy of micelles. Mice were randomly divided into six groups (six mice/group): saline, PTX, APA, PTX + APA, PA-NPs, and P-NPs. Different drug formulations with equivalent doses of PTX (5.0 mg/kg) and APA (1.0 mg/kg) were administered via tail vein injection on days 0, 3, and 6 after tumor sizes reached 50 mm^3^. Tumor volume and mouse body weight were measured every three days during the experiment. Our data showed that all the drug formulations exerted significant inhibitory effects on tumor growth, compared with the saline control group (Figure 6(C,E)). Additionally, PTX in combination with APA exerted a higher inhibitory effect than PTX alone, suggestive of reversal of MDR. As expected, PA-NPs exhibited the strongest inhibitory activity relative to the control group. Tumor inhibition rates (TIR) were determined as 27.1% in PTX, 48.2% in PTX + APA, 66.7% in P-NP, and 85.5% in PA-NP treatment groups (Figure 6(D)). Our H&E assay results further indicated massive cancer cell remission in tumor tissues treated with PA-NPs (Figure 6(G)). Thus, our strategy involving combination of active tumor-targeting drugs and P-gp inhibitors has remarkable potential in combating MDR.

To evaluate the safety of all drug formulations, the body weights of mice were further recorded. No obvious weight loss was observed in any of the treatment groups, indicating good biocompatibility (Figure 6(F)).

## 4. Conclusions

Here, we successfully developed a redox-sensitive polymer as a lipophilic carrier for co-delivery of PTX and APA to achieve reversal of MDR. In this system, PTX and APA were rapidly released from PA-NPs under conditions of high GSH levels, and their intracellular cytotoxicities and concentrations were significantly increased in drug-resistant MCF-7/ADR cells via HA-mediated active targeting of tumor cells and APA-induced inhibition of P-gp function. Moreover, the longer blood circulation times promoted drug accumulation in tumor sites, facilitating effective suppression of tumor growth. Our newly designed smart co-delivery system provides an effective option in the clinical treatment of MDR breast cancer.

## Supplementary Material

Supplemental Material
