# Supplementary material for: Hyaluronic acid reduction-sensitive polymeric micelles achieving co-delivery of tumor-targeting paclitaxel/apatinib effectively reverse cancer multidrug resistance
Source: Drug Deliv. 2020 Jun 3;27(1):825–35. doi: 10.1080/10717544.2020.1770373 (PMC8216478; doi:10.1080/10717544.2020.1770373)
Supplement: Supplemental Material [file IDRD_A_1770373_SM1089.docx]

**Supporting Information**

Hyaluronic acid reduction-sensitive polymeric micelles achieving co-delivery of tumor-targeting paclitaxel/apatinib effectively reverse cancer multidrug resistance

Xiaoqing Zhang1#, Xiaomei Ren1#, Jiayin Tang1, Jiangtao Wang1, Xiang Zhang2, Peng He1, Chang Yao1, Weihe Bian1, Lizhu Sun2*

1. Department of Mastopathy, The Affiliated Hospital of Nanjing University of Chinese Medicine (Jiangsu Province Hospital of TCM), Nanjing 210029, China.

2. The Department of Oncology, The Affiliated Shuyang Hospital of Xuzhou Medical University.

**Corresponding author:**

Lizhu Sun, Department of Oncology, Shuyang Hospital Affiliated to Xuzhou Medical University, Shuyang People's Hospital, Suqian, 223600, China

* E-mail: [dorslz@163.com](mailto:dorslz@163.com),

**# These authors contributed equally to this manuscript.**

**Supporting materials and experiments**

**1. Materials**

Hyaluronic acid (HA, molecular weight: 3.0 kDa) was obtained from Shanghai YuanYe Biotechnology co., Ltd (Shanghai, China). The *N*ε-benzyloxycarbonyl-L-lysine-*N*-carboxyanhydride (Lys-NCA) was synthesized according to previously mentioned methods.^[1,2]^ Paclitaxel (PTX), apatinib (APA), Nile red, Rhodamine 123 (Rh123), verapamil (VRP), cystamine dihydrochloride (Cys), N-Hydroxysuccinimide (NHS), 1-(3-dimethylaminopropyl)-3-ethylcarbodiimide hydrochloride (EDC), and glutathione (GSH) were obtained from Aladdin Reagent Co. Ltd. (Shanghai, China). Triton X-100, -(4,5-dimethylthiazol-2-yl)-2,5-diphenyl tetrazolium bromide (MTT), DAPI, and BCA kit were purchased from Beyotime Institute of Biotechnology (Shanghai, China). P-gp antibody (ab242104), and GAPDH antibody (EPR16891) were obtained from abcam. For PTX treatment, 6.0 mg of PTX was dissolved in the mixture of Cremophor EL and ethyl alcohol (v/v: 1:1).

**2. Characterization**

The structure of HA-Cys and HA-ss-PLLZ was measured by ^1^H NMR spectra on a Bruker AVANCE III spectrometer at 300 MHz, and tetramethyl silane (TMS) was used as the internal standard. D_2_O or DMSO-*d6* was used as the solvent depending on polymer solubility. Size distribution and zeta potential were determined by dynamic light scattering (DLS, Zetasizer Nano Zs90, Malvern). The morphology of nanoparticles was measured by a transmission electronic microscopy (TEM, Tecnai G2 20 WTWIN, Philips). PTX and APA content in different micelles was measured by a HPLC method using a C18 column (Agilent ODS C18 column, 4.6 × 250 mm, 5 μm particle size) in a Shimadzu HPLC system. For PTX analysis, the mobile phase was set at 35% acetonitrile and 65% water, and the detection wavelength at 227 nm. For APA analysis, the mobile phase was set at 33% acetonitrile and 67% water (contented 1% TEA), and the detection wavelength at 260 nm. Molecular weight HA-ss-PLLZ was determined by gel permeation chromatography (GPC) using a Waters GPC system1 (Waters Styragel HT6E column, with OPTILAB DSP interferometric refractometer as the detector). Formamide was used as the eluent at a flow rate of 1.0 ml/min at 40 °C. Poly(ethylene glycol) with different molecular weights were used to generate the calibration curves.

**3. Cell lines and animals**

The human breast cancer cell line MCF-7 cells and MCF-7/ADR cells were obtained from the Institute of Biochemistry and Cell Biology, Shanghai Institutes for Biological Sciences, Chinese Academy of Sciences (Shanghai, China) and Keygen Biotech. Co., Ltd. (Nanjing, China), respectively. The MCF-7 and MCF-7/ADR cells were cultured in RPMI 1640 culture medium, containing 10% (v/v) fetal bovine serum and 100 IU/mL penicillin and 100 µg/mL streptomycin at 37 °C in a humidified 5% CO_2_ atmosphere. Drug resistance of MCF-7/ADR was maintained by the addition of doxorubicin (1 µg/mL) in the medium. Before assay, the cells were cultured in drug-free medium for 15 days.

BALB/c nude mice (female, 4-6 weeks, 20.0 ± 2.0 g) and SD rates (male, 3-5 weeks, 120.0 ± 13.0 g) were purchased from the Vital River Laboratory Animal Technology Co., Ltd. (Beijing, China). All animals received care in compliance with the guidelines outlined in the Guide for the Care and Use of Laboratory Animals. All procedures were approved by the TCM Care and Use Committee of the Jiangsu Province Hospital.

**4.** **Critical micelle concentrations (CMC) determination of HA-ss-PLLZ**

Nile red was employed as fluorescence probe to detect CMC values of the HA-ss-PLLZ. ^[3,4]^ HA-ss-PLLZ was dissolved in PBS to designated the concentration (rang 0.25-120.00 µg/mL). Then DMSO solution of Nile red was added to a final concentration 6.0 × 10^-7^ M. Fluorescence spectrometer (λex = 557 nm, λem = 601 nm) was performed to detected the fluorescence intensity of the solutions. Subsequently, the CMC value of HA-ss-PLLZ was measured by extending the liner fluorescence intensity of both the high and low concentration region.

**5. Stability test of PA-ss-NPs**

The PA-ss-NPs solution (5 mg/mL) was prepared and mixed with PBS or without FBS. The content of FBS was fixed at 20% (v/v). The mixture was incubated at 37 °C and the size of PA-ss-NPs tested at intervals using DLS.

**6. Western blot analysis**

P-gp expression was measured by western blot analysis as previously described.^[5]^

**7. Hemolysis assay**

The hemocompatibility of P-NPs and PA-NPs at different concentrations was assessed by hemolysis assay.^[6]^ Typically, fresh mouse blood was diluted by PBS (pH 7.4), and red blood cells (RBCs) were collected by centrifugation. The RBCs were further diluted by PBS, and then added PA-NPs and P-NPs at various concentrations (0.15, 0.3, 0.6, 1.25, 2.5, 5.0, and 10.0 mg/mL). The mixture was cultured at 37 °C for 3 h and the released of hemoglobin was recorded on a microplate plate reader at 540 nm. Both PBS and distilled water was employed as negative and positive control. Hemolysis ratio (HR) was calculated according to the following formula:

Hemolysis (%) = (As – An) / (Ap-An) × 100. “As” is presented sample’s absorbance, “Ap” and “An” are presented the positive control and negative control group’s absorbance, respectively.

**Supporting figures and tables**





**Fig. S1** Synthesis rout of HA-ss-PLLZ


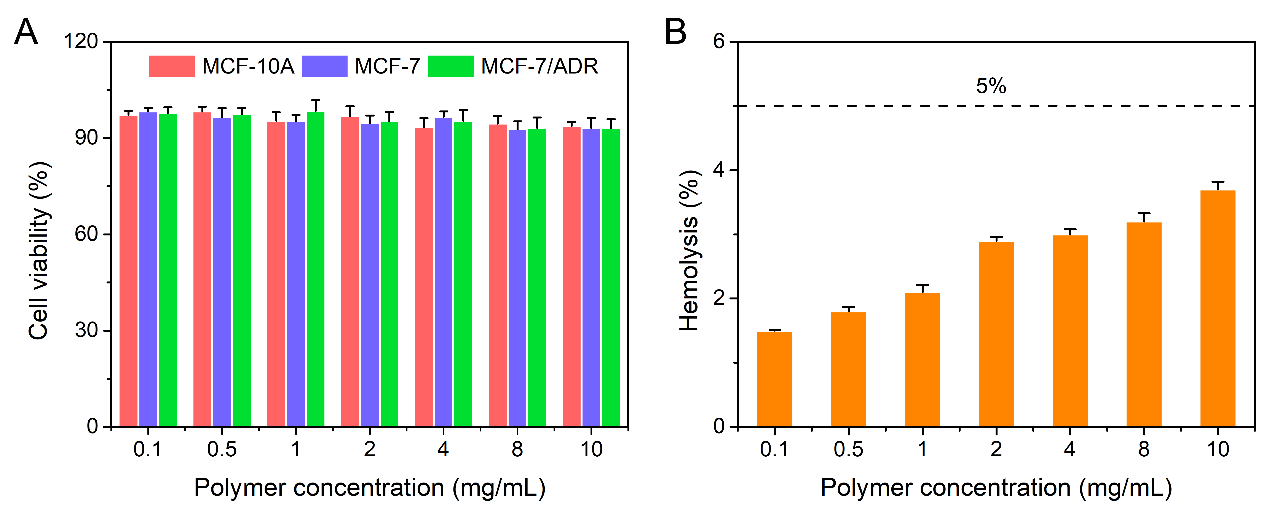


**Fig. S2** HA-ss-PLLZ biocompatibility analysis. (A) MTT results of HA-ss-PLLZ against to two breast cancer cell lines (MCF-7/ADR and MCF-7 cells) and one normal breast cell line (MCF-10A) (*n* = 6). (B) Hemolysis test of HA-ss-PLLZ against mouse RBCs (*n* = 3).


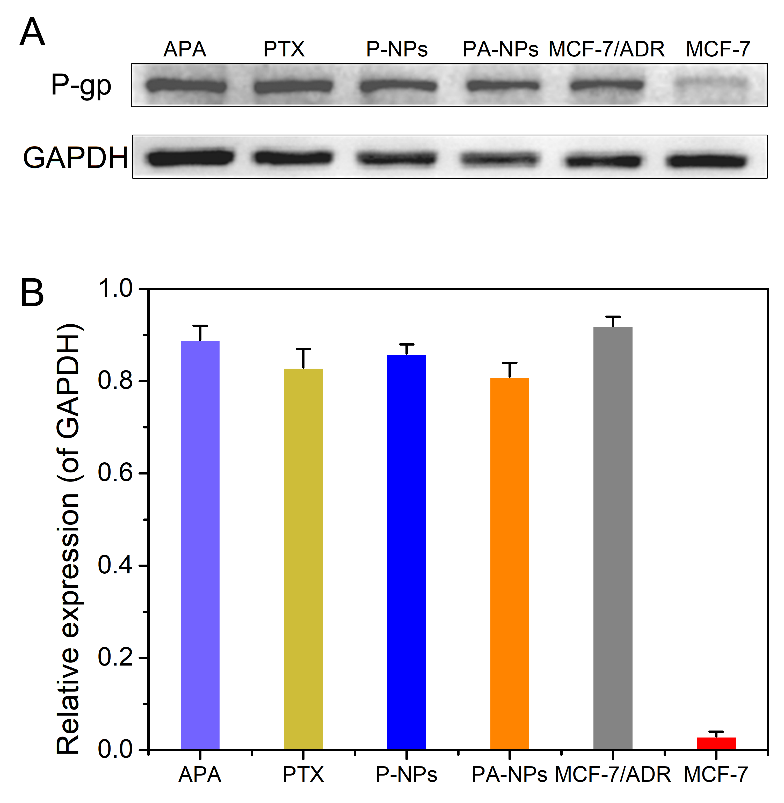


**Fig. S3** Western blotting analysis P-gp expression level on MCF-7/ADR and MCF-7 cells, as well as the affect of APA, PTX, P-NPs, and PA-NPs in its expression on MCF-7/ADR cells.

| **Table S1**. Characterization of HA-ss-PLLZ | | | | | |
| --- | --- | --- | --- | --- | --- |
| Polymer  (HA-ss-PLLZ_n_) | Mn^a^ (Da) | Mn^b^ (Da) | CMC (μg/mL) | Size (nm) | PDI |
| HA-ss-PLLZ_5_ | 5,127 | 5,011 | Very soluble | | |
| HA-ss-PLLZ_10_ | 7,213 | 7,089 | 98.7 | 345.7 ± 8.5 | 0.32 ± 0.01 |
| HA-ss-PLLZ_15_ | 9,334 | 9,233 | 22.8 | 217.5 ± 4.9 | 0.27 ± 0.02 |
| HA-ss-PLLZ_20_ | 11,162 | 11,587 | 6.1 | 91.4 ± 4.3 | 0.18 ± 0.03 |
| HA-ss-PLLZ_25_ | 13,258 | 12,356 | 5.7 | 123.4 ± 3.5 | 0.35 ± 0.04 |
| HA-ss-PLLZ_30_ | 15,377 | 15,646 | Insoluble in PBS | | |

**Note:** 1) Subscript _n_ in the HA-ss-PLLZ_n_ means the amount of PLLZ in polymer. 2) ^a^ Estimated by 1H NMR. PLL amount was calculated by comparing the signal intensities of benzyl protons with protons of HA from 3.0-4.0 ppm. 3) ^b^ Detected by GPC. Da: Dalton. PDI: polydispersity index.

| **Table. S2** Characterization of drug-loaded micelles. | | | | | | | |
| --- | --- | --- | --- | --- | --- | --- | --- |
| Micelles | Size (nm) | PDI | Zeta potential (mV) | LC (%) | | EE (%) | |
|  |  |  |  | PTX | APA | PTX | APA |
| PA-NPs | 122.3±3.1 | 0.21±0.03 | -15.3±0.8 | 13.5±0.9 | 3.4±0.3 | 80.2±2.4 | 61.3±4.1 |
| P-NPs | 109.2±1.9 | 0.19±0.02 | -16.2±0.7 | 14.7±1.1 | - | 87.6±3.5 | - |

**References**

[1] Christie RJ, Yu M, Miyata K, et al. Targeted Polymeric Micelles for siRNA Treatment of Experimental Cancer by Intravenous Injection. *Acs Nano* 2012;6:5174.

[2] Wei-Hai C, Guo-Feng L, Qi L, et al. MMP-2 responsive polymeric micelles for cancer-targeted intracellular drug delivery. *Chemical Communications* 2014;51:465-8.

[3] Li J, Xu R, Lu X, et al. A simple reduction-sensitive micelles co-delivery of paclitaxel and dasatinib to overcome tumor multidrug resistance. *International journal of nanomedicine* 2017;12:8043-56.

[4] Sun J, Liu Y, Chen Y, et al. Doxorubicin delivered by a redox-responsive dasatinib-containing polymeric prodrug carrier for combination therapy. *Journal of controlled release : official journal of the Controlled Release Society* 2017;258:43-55.

[5] Tang H, Zeng L, Wang J, et al. Reversal of 5-fluorouracil resistance by EGCG is mediate by inactivation of TFAP2A/VEGF signaling pathway and down-regulation of MDR-1 and P-gp expression in gastric cancer. *Oncotarget* 2017;8:82842.

[6] Moghimipour E, Rezaei M, Ramezani Z, et al. Folic acid-modified liposomal drug delivery strategy for tumor targeting of 5-fluorouracil. *European journal of pharmaceutical sciences : official journal of the European Federation for Pharmaceutical Sciences* 2018;114:166-74.
